# Outcomes and cost analysis of patients with dementia in the intensive care unit: a population-based cohort study

**DOI:** 10.1186/s12913-023-10095-5

**Published:** 2023-10-19

**Authors:** C. Dziegielewski, SM. Fernando, C. Milani, R. Mahdavi, R. Talarico, LH. Thompson, P. Tanuseputro, K. Kyeremanteng

**Affiliations:** 1 Department of Medicine, University of Ottawa, Ottawa, ON Canada; 2 Division of Critical Care, Department of Medicine, University of Ottawa, Ottawa, ON Canada; 3 Department of Critical Care, Lakeridge Health Corporation, Oshawa, ON Canada; 4 ICES, University of Ottawa, Ottawa, ON Canada; 5 Bruyere Research Institute, Ottawa, ON Canada; 6 Division of Palliative Care, Department of Medicine, University of Ottawa, Ottawa, ON Canada; 7 Clinical Epidemiology Program, Ottawa Hospital Research Institute, Ottawa, ON Canada

**Keywords:** Dementia, Intensive care, Acute care, Costs, Healthcare expenditure

## Abstract

**Background:**

Dementia is a neurological syndrome affecting the growing elderly population. While patients with dementia are known to require significant hospital resources, little is known regarding the outcomes and costs of patients admitted to the intensive care unit (ICU) with dementia.

**Methods:**

We conducted a population-based retrospective cohort study of patients with dementia admitted to the ICU in Ontario, Canada from 2016 to 2019. We described the characteristics and outcomes of these patients alongside those with dementia admitted to non-ICU hospital settings. The primary outcome was hospital mortality but we also assessed length of stay (LOS), discharge disposition, and costs.

**Results:**

Among 114,844 patients with dementia, 11,341 (9.9%) were admitted to the ICU. ICU patients were younger, more comorbid, and had less cognitive impairment (81.8 years, 22.8% had ≥ 3 comorbidities, 47.5% with moderate-severe dementia), compared to those in non-ICU settings (84.2 years, 15.0% had ≥ 3 comorbidities, 54.1% with moderate-severe dementia). Total mean LOS for patients in the ICU group was nearly 20 days, compared to nearly 14 days for the acute care group. Mortality in hospital was nearly three-fold greater in the ICU group compared to non-ICU group (22.2% vs. 8.8%). Total healthcare costs were increased for patients admitted to ICU vs. those in the non-ICU group ($67,201 vs. $54,080).

**Conclusions:**

We find that patients with dementia admitted to the ICU have longer length of stay, higher in-hospital mortality, and higher total healthcare costs. As our study is primarily descriptive, future studies should investigate comprehensive goals of care planning, severity of illness, preventable costs, and optimizing quality of life in this high risk and vulnerable population.

**Supplementary Information:**

The online version contains supplementary material available at 10.1186/s12913-023-10095-5.

## Background

Dementia is a common neurological syndrome that affects the aging population. It is estimated that there are nearly 6 million adults aged 65 and older are currently living with Alzheimer’s dementia in the United States [1]. This age group is expected to comprise 20% of the population by 2030, nearly doubled from 2010, which will lead to increased rates of dementia diagnoses [2, 3]. Patients with dementia utilize acute care hospital services twice as much as age-matched patients without dementia [3–7]. Several studies have demonstrated that patients with dementia have higher rates of ICU admissions, and it is expected that they will account for up to 25% of ICU admissions by the end of 2020 [8, 9]. Dementia incurs substantial costs to the healthcare system—upwards of $150 billion in a year in the U.S. With the aging population, ICU costs are expected to increase by over 80% by 2026 [10–13]. The compound of an increasingly older population with increasing rates of dementia diagnoses may result in an escalating demand for critical care and rising costs, which requires an evaluation of healthcare expenditure in this patient population.

While it has been shown that dementia results in increased healthcare costs, there is limited research evaluating ICU costs for patients with dementia. Previous population-based studies have described conflicting data on this, some suggesting dementia results in increased costs while others report reduced costs due to decreased length of stay [9, 14]. Most of these studies were conducted in smaller, institutional cohorts, and costs after admission were not investigated. Furthermore, there is limited literature available describing functional outcomes of patients with dementia after ICU stay. Critically ill patients with dementia often experience increased frailty post discharge, and are at increased risk of delirium and further cognitive decline, which highlights the necessity of establishing early goals of care and determining who may benefit from ICU stay [6, 8, 9, 15]. We sought to describe the characteristics and outcomes of patients with dementia in the ICU as well as non-ICU hospital settings, and perform a comprehensive cost analysis of both inpatient and outpatient costs after admission.

## Methods

Studies conducted at ICES (formerly the Institute for Clinical Evaluative Sciences) using administrative data fall under Sect. 45 of the Personal Health Information Protection Act of Ontario, and do not require research ethics board approval. Studies at ICES do not require informed consent and study data is anonymized before its use.

### Data sources and setting

We conducted a retrospective observational population-based cohort study in Ontario, Canada (population 14.6 million). Within Ontario’s single payer healthcare system, all publicly funded healthcare services, physician, hospital, and demographic information for residents are recorded in these databases. These datasets were linked using unique encoded identifiers, and analysed at ICES, an independent, non-profit research institute whose legal status under Ontario’s health information privacy law allows it to collect and analyse healthcare and demographic data, without consent, for health system evaluation and improvement [16]. Data contained in ICES is complete, with the exception of emigration from Ontario, which represents approximately 0.5% of patients per year [17]. Databases were linked and then anonymized at the individual level at ICES (Additional File Table 1).

### Patient population

The study population included all patients aged 65 years or older with a previous dementia diagnosis and a hospital stay from April 1, 2016 to March 31, 2019. We identified adult patients admitted to an ICU setting during the study period by using previously validated algorithms from the Canadian Institute for Health Information Discharge Abstract Database (DAD) and methods used by Scales et al. [18–20]. There has been consistency among hospitals in Ontario, with specificity of greater than 95% for the majority of hospitals studied [20]. We also identify patients not requiring an ICU admission (non-ICU group). For patients with multiple ICU admissions within this timeframe, only the first ICU admission was included. Transfers to different hospitals were included in the same episode of care. Patients were excluded if they were younger than 65 years of age or older than 105 years at the time of the index assessment, the date of admission or date of discharge were missing, or if they were not Ontario Health Insurance Plan (OHIP) eligible during hospital admission or follow-up.

Comorbid conditions were presented using the Charlson comorbidity score, a score calculated based on a list of medical conditions a patient has within hospital records [21]. We identified complex chronic diseases among our cohort, using previously described methods [18]. All other conditions were based on the presence of any one inpatient hospital diagnostic code, or two or more outpatient physician billing codes within a 2-year period, using relevant ICD, Version 9 (ICD-9) and ICD-10 codes (Additional File Table 2). We assessed palliative care involvement using previously validated methods by Webber et al., in which patients either had a palliative care diagnosis, admission to palliative care service, or involvement of a palliative care specialist based on billings claims during their hospitalization [22]. Outpatient and palliative care home services would not be captured by this algorithm.

We identified patients with dementia using the following criteria: (1) diagnosis of dementia in a previous hospitalization (obtained from the DAD), or (2) three or more physician billing claims at least 30 days apart in a two-year period (obtained from the OHIP claims database), or (3) prescription of a cholinesterase inhibitor (obtained from the ODB database), or (4) documentation of dementia or Alzheimer’s disease AND Cognitive Performance Scale (CPS) score greater than or equal to 2 in index assessment or in any previous RAI assessment, including those administered for complex continuing care and long-term care services (CCRS database) and home care services (RAI-HC database). These criteria have been previously shown to have high positive predictive value and have been applied in several studies [23, 24]. For patients who had dementia and underwent CPS and RAI assessment in complex continuing care, long-term care, or home care, severity of cognitive impairment, including functional impairment in activities of daily living (ADLs) and instrumental activities of daily living (IADLs), were described (Additional File Table 5).

### Outcome variables

The primary outcome variable was mortality. Secondary outcomes included ICU and hospital LOS, discharge disposition, hospital readmissions, and healthcare costs. Patients were also followed up for up to one year post-index admission to determine if there were recurrent ED visits, or re-admissions to hospital or ICU. The healthcare visits post-index admission were censored for death. We determined discharge disposition using a hierarchy approach (Additional File Table 6).

We obtained the total and sector-specific direct healthcare costs accumulated in the year following the date of the index ICU admission (including the admission itself). As all healthcare costs are absorbed by OHIP, these were records of healthcare paid for by the Ontario Ministry of Health (MOH). We estimated the costs associated with each record using previously described costing guidelines [25]. Briefly, we’ve taken a payer (MOHLTC) costing perspective, using person-level health care expenditures that accounts for data for health care utilization and cost information per use. Cost information for sectors (e.g., hospitals, complex continuing care, rehab) that have global budgets (e.g., by institution or by health region) were determined using a top-down approach through case-mix methodology. Sectors that have fee payments associated with each use (e.g., drug cost, or cost paid out to physician) had costs estimated directly. These costs included index hospital admission up to one year after admission. We expressed all costs in 2020 Canadian dollars, and past costs were adjusted for inflation using the yearly Consumer Price Index reported by Statistics Canada [26].

### Statistical analysis

We conducted statistical analysis using SAS Enterprise Guide 7.1 (SAS Institute Inc., Cary, NC, USA). We presented descriptive statistics as percentages, mean (with standard deviation), or median (with interquartile range), as appropriate. We used chi-square test or Fisher’s exact test (categorical variables) or Mann-Whitney U test (parametric variables). We used logistic regression to model hospital survival as an outcome in the total patient cohort. We also used logistic regression to model ICU admission in the patient cohort that had data on severity of cognitive impairment and functional status available. The predictor variables of interest were age, sex, income quintile, Charlson score, number of ED and hospital visits before the index admission, as well as the presence of the most prevalent comorbidities. Results are presented as odds ratios (OR) with 95% confidence intervals (CI). P-values of < 0.05 were considered statistically significant.

## Results

We identified a total of 114,844 patients with dementia who met inclusion criteria. Of these, 11,341 (9.9%) were admitted to the ICU and 103,503 (90.1%) were admitted to non-ICU hospital settings. Baseline characteristics for both patient groups are described in Table 1. Patients were younger in the ICU, compared to those outside of the ICU (mean age 81.8 [7.53] years vs. 84.2 [7.57] years, *p* < 0.001). The Charlson comorbidity index score was ≥ 3 in 22.8% of patients in the ICU group, and 15.0% in the non-ICU group (*p* < 0.001).

Outcome variables are listed in Table 2. Mean total hospital LOS for patients was greater in the ICU group, compared to the non-ICU group (19.6 [43.2] vs. 14.0 [34.8], *p* < 0.001). There was no significant difference in delirium between the two groups (18.9% vs. 19.1%, *p* = 0.62). In the ICU group, 26.6% of patients were mechanically ventilated, compared to 0.4% in the non-ICU group (*p* < 0.001). There were higher rates of death in hospital in the ICU group compared to non-ICU group (22.2% vs. 8.8%, *p* < 0.001). Survival at one-year post-discharge was 55.8% in the ICU group, compared to 63.7% in the non-ICU group (*p* < 0.001). For the ICU group, 62.9% were discharged to a disposition other than home without homecare, in comparison to 76.4% for the non-ICU group (*p* < 0.001). Notably, less than 15% of patients in both groups were discharged home without homecare (14.9% for ICU vs. 14.8%, for non-ICU group, *p* < 0.001). There was more time spent at home after discharge from the ICU rather than discharge from non-ICU hospital settings (mean 192.7 [162.7] days vs. 178.3 [161.5] days, *p* < 0.001). Nearly twice as many patients (9.9% vs. 5.0%, *p* < 0.001) were re-admitted to the ICU after index hospitalization.

**Table 1:** Baseline characteristics of patients with dementia admitted to the ICU or non-ICU hospital settings

Variable	ICU (*n* = 11,341)	Non-ICU (*n* = 103,503)	P value
Age, mean (SD)	81.8 (7.53)	84.2 (7.57)	< 0.001
Age categories, n (%)			
65–75 76–85 86–95 95+	2,490 (22.0%) 4,974 (43.9%) 3,622 (31.9%) 255 (2.2%)	14,933 (14.4%) 39,720 (38.4%) 43,928 (42.4%) 4,922 (4.8%)	< 0.001
Male, n (%)	5,506 (48.5%)	42,885 (41.4%)	< 0.001
Neighbourhood income quintile, n (%)			
Lowest Low Middle High Highest Missing	3,012 (26.6%) 2,451 (21.6%) 2,254 (19.9%) 1,837 (16.2%) 1,716 (15.1%) 71 (0.6%)	27,025 (26.1%) 23,141 (22.4%) 19,295 (18.6%) 17,258 (16.7%) 16,176 (15.6%) 608 (0.6%)	0.011
Rurality, n (%)			
Urban Rural Missing	9,894 (87.2%) 1,379 (12.2%) 68 (0.6%)	92,284 (89.2%) 10,642 (10.3%) 577 (0.6%)	< 0.001
Years since dementia diagnosis, mean (SD)	3.30 (3.95)	3.35 (3.78)	0.20
Location prior to hospitalization			
Acute Care Hospitalization Rehab Hospitalization Psychiatric Hospitalization Palliative Care Hospitalization Complex Continuing Care Long-term Care Supportive/Transitional Housing Home Care Ambulatory Care Same Day Surgery Missing/Unknown	330 (2.9%) 57 (0.5%) 47 (0.4%) 1,195 (10.5%) 142 (1.3%) 1,530 (13.5%) 404 (3.6%) 44 (0.4%) 954 (8.4%) 5 (0.0%) 6,640 (58.5%)	1,528 (1.5%) 406 (0.4%) 255 (0.2%) 14,710 (14.2%) 770 (0.7%) 17,004 (16.4%) 5,195 (5.0%) 768 (0.7%) 4,038 (3.9%) 25 (0.0%) 58,798 (56.8%)	< 0.001
Charlson comorbidity index score, n (%)			
0–2 3+	8,753 (77.2%) 2,588 (22.8%)	88,023 (85.0%) 15,480 (15.0%)	< 0.001
Chronic conditions (by diagnosis), n (%)			
Hypertension Diabetes Osteoarthritis Cancer CHF CAD Arrhythmia Renal Failure COPD Stroke	5,194 (45.8%) 4,069 (35.9%) 2,961 (26.1%) 2,883 (25.4%) 2,031 (17.9%) 1,858 (16.4%) 1,500 (13.2%) 1,468 (12.9%) 1,457 (12.8%) 803 (7.1%)	44,738 (43.2%) 31,273 (30.2%) 26,975 (26.1%) 26,051 (25.2%) 15,082 (14.6%) 12,452 (12.0%) 11,547 (11.2%) 12,101 (11.7%) 9,914 (9.6%) 6,232 (6.0%)	< 0.001 < 0.001 0.01 < 0.001 < 0.001 < 0.001 < 0.001 < 0.001 < 0.001 0.016

Notes: Age is represented by mean years (SD). Rest of the data is represented by n (%), where n=number of patients. CAD=coronary artery disease; CKD=chronic kidney disease; CHF=congestive heart failure; COPD=chronic obstructive pulmonary disease; MI=myocardial infarction

**Table 2:** Outcome variables for patients with dementia admitted to the ICU or non-ICU hospital settings

Variables	ICU (*n* = 11,341)	Non-ICU (*n* = 103,503)	P value
ICU Length of stay, mean (SD)	4.65 (11.7)	N/A	< 0.001
Acute Care Length of stay, mean (SD)	13.6 (21.2)	7.82 (10.4)	< 0.001
Total Hospital Length of stay, mean (SD)	19.6 (43.2)	14.0 (34.8)	< 0.001
ALC Length of stay, mean (SD)	6.05 (32.8)	6.12 (31.4)	0.81
Palliative care involvement, n (%)	112 (1.0%)	1,766 (1.7%)	< 0.001
Delirium, n (%)	2,140 (18.9%)	19,728 (19.1%)	0.62
Procedures/interventions, n (%)			
Invasive mechanical ventilation Dialysis Feeding tube Bronchoscopy CPR Non-invasive mechanical ventilation Defibrillation PCI	3,021 (26.6%) 311 (2.7%) 543 (4.8%) 206 (1.8%) 309 (2.7%) 523 (4.6%) 150 (1.3%) 485 (4.3%)	392 (0.4%) 762 (0.7%) 632 (0.6%) 60 (0.1%) 136 (0.1%) 280 (0.3%) 56 (0.1%) 363 (0.4%)	< 0.001 < 0.001 < 0.001 < 0.001 < 0.001 < 0.001 < 0.001 < 0.001
Discharge Disposition, n (%)			
Discharged to CCC/Rehab Discharged to LTC Discharged to Home with Homecare Discharged to Home without Homecare Death in Hospital	1,515 (13.4%) 2,056 (18.1%) 3,566 (31.4%) 1,687 (14.9%) 2,517 (22.2%)	11,325 (10.9%) 24,727 (23.9%) 43,018 (41.6%) 15,295 (14.8%) 9,138 (8.8%)	< 0.001
Survival, n (%)			
Survival at discharge 3 month survival post discharge 6 month survival post discharge 12 month survival post discharge	8,824 (77.8%) 7,556 (66.6%) 7,071 (62.3%) 6,332 (55.8%)	94,365 (91.2%) 80,482 (77.8%) 74,833 (72.3%) 65,899 (63.7%)	< 0.001 0.39 0.06 < 0.001
Home time, mean (SD)			
Days at home Days at an institution Total number of days	192.7 (162.7) 103.2 (138.5) 295.9 (123.9)	178.3 (161.5) 114.7 (143.4) 293.0 (124.8)	< 0.001 < 0.001 < 0.001
Hospital admissions pre-index admission, n (%)			
0 1 2+	8,895 (78.4%) 1,583 (14.0%) 863 (7.6%)	81,381 (78.6%) 14,702 (14.2%) 7,420 (7.2%)	< 0.001
ED visits pre-index admission, n (%)			
0 1 2+	3,873 (34.2%) 3,330 (29.4%) 4,138 (36.5%)	29,272 (28.3%) 31,069 (30.0%) 43,162 (41.7%)	< 0.001
Hospital re-admissions post-index admission, n (%)			
0 1 2+	7,368 (65.0%) 2,413 (21.3%) 1,560 (13.8%)	63,588 (61.4%) 24,903 (24.1%) 15,012 (14.5%)	<0.001
ICU re-admissions post-index admission, n (%)			
0 1 2+	10,216 (90.1%) 944 (8.3%) 181 (1.6%)	98,408 (95.1%) 4,626 (4.5%) 469 (0.5%)	< 0.001
Outpatient admissions post-index admission, n (%)			
0 1 2+	10,195 (89.9%) 852 (7.5%) 294 (2.6%)	93,427 (90.3%) 7,269 (7.0%) 2,807 (2.7%)	< 0.001
Number of ED visits post-index admission, n (%)			
0 1 2+	5,744 (50.6%) 2,239 (19.7%) 3358 (29.6%)	45,989 (44.4%) 23,948 (23.1%) 33,566 (32.4%)	0.07

Notes: Length of stay (LOS) is represented by mean days (standard deviation). Acute Care LOS is defined as time spent in hospital outside of ALC (alternative level of care) days. Total hospital LOS is the sum total acute care LOS and ALC LOS. A one-year lookback period before index admission date is used to determine the number of hospital admissions or ED visits pre-index admission. A one-year follow up period after index admission date is used to determine the number of ICU re-admissions, hospital re-admissions or ED visits post-index admission. PCI=percutaneous coronary intervention; CPR=cardiopulmonary resuscitation; CCC=complex continuing care; LTC=long term care; ED=emergency department

Data on severity of cognitive impairment and functional status was available for 5,571 (49.1%) of patients in the ICU group, and 60,325 (58.3%) of patients in the non-ICU group. Severity of cognitive impairment and functional status for patients in both groups is outlined in Table 3. There were more patients with moderate-severe cognitive impairment in the non-ICU group, compared to the ICU group (54.1% vs. 47.5%, *p* < 0.001). There were more patients with severe functional impairment in IADLs in the ICU group (39.4% vs. 19.6%, *p* < 0.001), but less severe impairment in ADLs, compared to the non-ICU group (17.3% vs. 17.7%, *p* = 0.022).

**Table 3:** Severity of cognitive impairment and functional status for patients with dementia admitted to the ICU or non-ICU hospital settings

Variables	ICU (*n* = 5,571)	Non-ICU (*n* = 60,325)	P value
Severity of cognitive impairment, n (%)			
Minimal Moderate Severe Missing	2,923 (52.5%) 1,955 (35.1%) 693 (12.4%) < 5 (0.0%)	27,697 (45.9%) 22,534 (37.4%) 10,092 (16.7%) 0 (0.0%)	< 0.001
ADLs, n (%)			
Independent Minimal assistance Extensive assistance Dependent Missing	905 (16.2%) 1,485 (26.7%) 2,217 (40.0%) 964 (17.3%) < 5 (0.0%)	8,853 (14.7%) 16,533 (27.5%) 24,253 (40.2%) 10,684 (17.7%) 0 (0.0%)	0.022
IADLs, n (%)			
No difficulty Minimal difficulty Significant difficulty Missing	18 (0.3%) 129 (2.3%) 2,193 (39.4%) 3,231 (58.0%)	148 (0.2%) 1,399 (2.3%) 11,842 (41.7%) 33,619 (55.7%)	< 0.001
CHESS scale, n (%)			
No health instability Mild-moderate instability Severe health instability Missing	1,522 (27.3%) 3,702 (66.5%) 347 (6.2%) < 5 (0.0%)	15,235 (25.3%) 41,374 (68.6%) 3,711 (6.2%) 0 (0.0%)	< 0.001

Notes: Dementia severity and ADL, IADL, and CHESS scoring defined in Additional File Table 5

Cost analysis is summarized in Table 4. Mean inpatient hospital costs were significantly higher in the ICU group, compared to the non-ICU group ($34,660 [$52,870] vs. $20,506 [$28,358], *p* < 0.001). Mean long-term care costs were higher in the non-ICU group, in comparison to the ICU group ($12,772 [$20,024] vs. $8,798 [$17,607], *p* < 0.001). Mean total healthcare costs were increased for patients admitted to ICU vs. those admitted outside of the ICU ($67,201 [$70,777] vs. $54,080 [$46,141], *p* < 0.001). Patients with dementia admitted to the ICU accounted for 12.0% of total healthcare costs, equivalent to approximately $762 million in total healthcare expenditure.

**Table 4:** Cost breakdown for patients with dementia admitted to the ICU or non-ICU hospital settings

Variables	ICU (*n* = 11,341)	Non-ICU (*n* = 103,503)	P value
Inpatient Hospital Costs, mean (SD)	$34,660 ($52,870)	$20,506 ($28,358)	< 0.001
ED Costs, mean (SD)	$1,060 ($1,002)	$1,051 ($1,051)	< 0.001
Same Day Surgery Costs, mean (SD)	$357 ($1,444)	$183 ($857)	0.37
CCC Costs, mean (SD)	$4,689 ($23,030)	$3,707 ($18,784)	< 0.001
LTC Costs, mean (SD)	$8,798 ($17,607)	$12,772 ($20,024)	< 0.001
Rehab Costs, mean (SD)	$1,996 ($8,225)	$1,634 ($7,136)	< 0.001
Home Care Services Costs, mean (SD)	$3,620 ($8,116)	$4,651 ($8,777)	< 0.001
Hospital Outpatient Clinic Costs, mean (SD)	$1,088 ($1,530)	$897 ($1,256)	< 0.001
GP Billing Costs, mean (SD)	$1,130 ($1,360)	$1,264 ($1,359)	< 0.001
Specialist Billing Costs, mean (SD)	$5,249 ($5,526)	$2,728 ($3,016)	< 0.001
Non-physician Billing Costs, mean (SD)	$14 ($57)	$15 ($56)	0.55
OHIP Lab Costs, mean (SD)	$145 ($201)	$166 ($204)	< 0.001
ODB Drug Costs, mean (SD)	$2,285 ($4,215)	$2,430 ($4,079)	< 0.001
Total Healthcare Costs, mean (SD)	$67,201 ($70,777)	$54,080 ($46,141)	< 0.001

Notes: All costs are represented as mean (SD). Costs are accumulated in the year following the date of the index admission (including the admission itself). All costs are expressed in CDN ($), adjusted to 2021 prices. ED=emergency department; OHIP=Ontario Health Insurance Plan; ODB=Ontario Drug Benefit; CCC=complex continuing care; LTC=long term care; GP=general practitioner

Multivariate logistic regression analysis was performed for hospital survival in the entire patient cohort (Additional File Table 7). Being female was associated with a higher likelihood of survival (OR 1.34, CI 1.29–1.39). Conversely, being ≥ 95 in age (OR 0.31, CI 0.28–0.34), admitted to ICU (OR 0.33, CI 0.31–0.34), and having a Charlson score of ≥ 3 (OR 0.39, CI 0.37–0.41) were all factors associated with a lower likelihood of survival. Patients also had a higher likelihood of survival with comorbidities including hypertension (OR 1.27, CI 1.22–1.33), diabetes (OR 1.26, CI 1.21–1.32), and CAD (OR 1.29, CI 1.21–1.38). Subset multivariate logistic regression analysis was performed for patients with data on severity of cognitive impairment and functional status available, with ICU admission as the outcome (Additional File Table 8). Patients had a lower likelihood of being admitted to ICU if female (OR 0.89, CI 0.84–0.94), or had moderate-severe cognitive impairment (OR 0.78, CI 0.73–0.82). Having a Charlson score of ≥ 3 (OR 1.67, CI 1.56–1.79) was associated with a higher likelihood of ICU admission. Patients also had a higher likelihood of ICU admission if comorbid with CHF (OR 1.28, CI 1.18–1.38) or CAD (OR 1.13, CI 1.03–1.23), and if they have a prior hospital admission (OR 1.09, CI 1.05–1.13). Age- and sex-stratified 1-year mortality in described in Additional File Table 9. With mortality assessed for only the ICU cohort (Additional File Tables 10 and 11), we find that being ≥ 95 in age (OR 2.35, CI 1.91–2.89), having a Charlson score of ≥ 3 (OR 1.50, CI 1.37–1.63), and moderate-severe cognitive impairment (OR 1.35, CI 1.25–1.45) were all factors associated with a higher likelihood of mortality in those who had an RAI assessment.

## Discussion

In this retrospective population-based cohort study, we describe the characteristics, outcomes, and cost patterns of patients with dementia admitted to the ICU and non-ICU hospital settings. Patients with dementia admitted to the ICU are younger, more comorbid, and less likely to be transferred from home or assisted living institutions. They have a longer total LOS, increased interventions, and higher mortality. They have less severe baseline impairment or functional impairment post-discharge. They were more frequently readmitted to ICU. Patients with dementia admitted to the ICU, despite representing less than 10% of the cohort, incurred higher total healthcare costs, totalling $762 million for our cohort.

We characterize patients with dementia who are admitted to the ICU as younger, more comorbid, more functionally impaired but with less severe cognitive impairment, as compared to those admitted to non-ICU hospital settings. However, they had higher in-hospital mortality, and nearly 50% mortality one year after discharge. Taken together, these findings may suggest that patients with dementia admitted to the ICU are frailer than those outside of the ICU, but had a higher severity of illness resulting in increased mortality. This is in keeping with the reported literature [27, 28]. We note that there was no significant difference in income status and rurality between the cohorts, suggesting these social determinants of health do not seem to impact who is admitted to the ICU setting. The higher mortality and increased functional impairment and frailty in patients with dementia in the ICU may suggest against offering aggressive medical management. However, we note these patients have lower rates of cognitive impairment, suggesting they may have been risk-stratified prior to admission as to who would benefit from ICU care. We note that more severe cognitive impairment in the ICU cohort is associated with increased mortality, and should be considered in the process of evaluating who may benefit from more aggressive medical management. The decision of offering ICU care also needs to be considered in the context of other variables that are associated with increased mortality, which in our study included older age, male sex, and multi-morbidity, consistent with previous studies and closely interlinked with frailty [29, 30]. In our study, there was a very low rate of palliative care involvement in both cohorts (< 2%); however, this primarily reflects palliative care admissions rather than consultations, the latter of which is often a source of advanced care planning. Additionally, perhaps goals of care discussions took place prior to hospitalization, or outside of formal palliative care involvement, given the well-known repercussions of increased mortality, frailty, and worsened quality of life in patients with dementia [31]. However, we found that patients were more likely to be admitted to the ICU if they had a prior hospital admission, and nearly twice as likely to be re-admitted to the ICU. Goals of care discussions have the potential to optimize quality of care while reducing futile care, and are particularly important in this vulnerable population [32]. Further research to stratify which patients with dementia have increased survival benefit and may benefit from ICU admission is necessary, as well as the impact of advanced care planning on ICU admissions in this population.

We demonstrate that patients with dementia who are critically ill admitted to the ICU incur increased costs in comparison to those admitted to non-ICU hospital settings. For our cohort, this is likely in part driven by increased total LOS and increased interventions seen in the patients admitted to ICU. Previous studies have described the impact of LOS and interventions such as invasive mechanical ventilation in prolonging LOS for patients in critical care [33–36]. Dispositioning costs were the second highest driver of costs overall. Costs were increased for critically ill patients needing rehabilitation and complex continuing care, but conversely higher for long-term care in the non-ICU group. Patients with dementia become frailer after hospitalization, therefore requiring costly dispositioning for increased supports in their post-discharge recovery [37]. As patients had more severe cognitive impairment in the non-ICU group, this may explain the need for institutionalization. This highlights the importance of increasing the number of community supports to improve flow in the hospital and reduce unnecessary acute care stay [38]. Furthermore, it represents the need for early goals of care discussion and palliative care involvement. Palliative care has been linked to reducing ICU LOS and costs, and early goals of care discussions have been associated with reduced mortality, ICU use, and hospitalization [39–41]. Future studies should explore strategies to mitigate costs while optimizing quality, patient-centered care for this vulnerable population.

This study has several limitations. Firstly, we utilized health administrative data which lacks certain clinical variables such as severity of illness or admission diagnoses, as well as social determinants of health such as ethnicity data. While we include comparative analysis, there are limitations in making certain conclusions given these lack of clinical variables, such as whether patients admitted to the ICU have higher acuity accounting for the decision to offer ICU care. The use of coding data for various diagnoses such as delirium likely underestimates the true incidence in our population, limiting interpretation of its impact in both ICU and non-ICU settings. We note a small proportion of patients in the non-ICU setting received invasive ventilation; this is likely because therapy was initiated in this setting and was coded as such, before the patient was ultimately transferred to the ICU for ongoing care, which affects analysis of these results. Furthermore, there is a lack of certain variables such as vasoactive medications to explain reasons for ICU admission. Location prior to hospitalization, dementia severity, and functional data was available for a subset of patients only, which limits our interpretation and generalizability of how these variables impact ICU admission. Furthermore, while palliative care involvement during hospitalization was available, information about goals of care discussions and palliative care involvement prior to hospital admission was unavailable, which would be tremendously valuable and better inform if early discussions influence ICU or non-ICU hospital admission in this vulnerable patient population. Cost data was available for several categories; however, separate ICU and non-ICU hospital costs, as well as other cost breakdowns such as procedural interventions and outpatient palliative care, were not obtainable, which could help identify major drivers of increased cost. Furthermore, while our cost data allows for some comparison between the cohorts, there are differences in baseline patient characteristics, and so implications per patient encounter must be interpreted with caution. Finally, the retrospective nature of this study allows association, but not causation, to be determined.

## Conclusions

Patients with dementia admitted to the ICU are younger, more comorbid, with longer LOS, increased interventions, and higher mortality, as compared to those admitted to non-ICU hospital settings. They have less severe cognitive and functional impairment post-discharge. Patients with dementia admitted to the ICU incurred higher total healthcare costs, as compared to those not admitted to the ICU. As our study is largely descriptive and has associated limitations, decisions regarding pursuing critical care should be comprehensive and include informed goals of care discussions with consideration of the patient’s frailty, severity of illness, comorbidities, and cognitive status. Given the increasing prevalence of dementia and escalating demands for critical care, future studies should investigate preventable costs and ways to optimize quality of life while reducing futile care in this patient population.

### Electronic supplementary material

Below is the link to the electronic supplementary material.

Supplementary Material 1

## Data Availability

All data generated or analyzed during this study are included in this published article [and its supplementary file].
