# Supplementary material for: Outcomes and cost analysis of patients with dementia in the intensive care unit: a population-based cohort study
Source: BMC Health Serv Res. 2023 Oct 19;23:1124. doi: 10.1186/s12913-023-10095-5 (PMC10588096; doi:10.1186/s12913-023-10095-5)
Supplement: Supplementary file 1 — Supplementary Material 1 [file 12913_2023_10095_MOESM1_ESM.docx]

**Additional Files**

**Outcomes and Cost Analysis of Patients with Dementia in the Intensive Care Unit: A Population-based Cohort Study**

Dziegielewski C^1^, Fernando SM^2,3^, Milani C^4^, Mahdavi R^4^, Talarico R^4^, Thompson LH^4^, Tanuseputro P^4,5,6,7^, Kyeremanteng K^2,6^

^1^Department of Medicine, University of Ottawa, Ottawa, Ontario, Canada

^2^Division of Critical Care, Department of Medicine, University of Ottawa, Ottawa, Ontario, Canada

^3^Department of Critical Care, Lakeridge Health Corporation, Oshawa, ON, Canada;

^4^ICES, University of Ottawa, Ottawa, Ontario, Canada

^5^Bruyere Research Institute, Ottawa, ON, Canada

^6^Division of Palliative Care, Department of Medicine, University of Ottawa, Ottawa, Ontario, Canada

^7^Clinical Epidemiology Program, Ottawa Hospital Research Institute, Ottawa, ON, Canada

Corresponding author: Claudia Dziegielewski, Department of Medicine, University of Ottawa, 451 Smyth Rd, Ottawa, ON, Canada, K1H 8M5. Email: cldziegielewski@toh.ca.

**Additional File Figure 1**: Cohort Creation Flow Chart.

All patients with a diagnosis of dementia with ICU or non-ICU hospital admission between April 1 2016 and March 31 2019

*n*=121,264

Exclude patients aged <65 and >105

*n*=115,982

Exclude patients with date of death on or before index admission

*n*=115,068

Exclude records with missing date of admission/discharge

*n*=115,050

Exclude patients not OHIP eligible consecutively from index admission until 1 year after admission

n=114,844

Patient inclusion list

*n*=114,844

*n*=1,062,617

Patients with non-ICU hospital admission

*n*=103,503

Patients with ICU admission

*n*=11,341

**Additional File Table 1**: Databases linked through ICES, on the basis of individual patient identifiers.

| **Database** | **Information Contained** |
| --- | --- |
| The Ontario Health Insurance Plan (OHIP) Claims Database | All data on physician fee-for-service claims for inpatient and outpatient services. |
| Canadian Institute for Health Information (CIHI) Discharge Abstract Database (DAD) | Data from all acute care hospitalizations, including detailed diagnostic and procedural information. |
| Registered Persons Database | Deaths and all demographic information. |
| Home Care Database | Publicly-funded homecare services. |
| National Ambulatory Care Reporting System (NACRS) | Data related to all Emergency Department use. |
| National Rehabilitation Reporting System | Data related to all inpatient rehabilitation programs. |
| Continuing Care Reporting System | Data on long-term care (e.g., nursing home) and complex continuing care (e.g., rehabilitation facility) use. |
| Statistics Canada Census | Data related to income quintile and rurality, through postal codes |
| Resident Assessment Instrument – Minimum Data Set 2.0 (RAI-MDS 2.0) | Data on dementia and functional status |

**Additional File Table 2**: Ontario Health Insurance Plan (OHIP), International Classification of Diseases, Version 9 (ICD-9), and Version 10 (ICD-10) diagnostic codes for categorization of comorbidities.

| **Condition [reference for validated algorithm]** | **ICD 9 / OHIP** | **ICD 10** | **ODB*** |
| --- | --- | --- | --- |
| Acute Myocardial Infarction (AMI) [1] | 410 | I21, I22 |  |
| Osteo- and other Arthritis:   1. Osteoarthritis 2. Other Arthritis (includes Synovitis, Fibrositis, Connective tissue disorders, Ankylosing spondylitis, Gout Traumatic arthritis, pyogenic arthritis, Joint derangement, Dupuytren’s contracture, Other MSK disorders) | 715  727, 729, 710, 720, 274, 716, 711,718, 728, 739 | M15-M19  M00-M03, M07, M10, M11-M14, M20-M25, M30-M36,  M65-M79 |  |
| Arthritis - Rheumatoid arthritis [2] | 714 | M05-M06 |  |
| Asthma [3] | 493 | J45 |  |
| Cancer | 140-239 | C00-C26, C30-C44, C45-C97 |  |
| Cardiac Arrhythmia | 427 (OHIP) / 427.3 (DAD) | I48.0, I48.1 |  |
| Congestive Heart Failure [4] | 428 | I500, I501, I509 |  |
| Chronic Obstructive Pulmonary Disease [5] | 491, 492, 496 | J41, J43, J44 |  |
| Coronary syndrome (excluding AMI) | 411-414 | I20, I22-I25 |  |
| Dementia [6] | 290, 331 (OHIP) / 046.1, 290.0, 290.1,  290.2, 290.3, 290.4, 294, 331.0, 331.1, 331.5, F331.82 (DAD) | F00, F01, F02, F03, G30 | Cholinesterase  Inhibitors |
| Diabetes [7] | 250 | E08-E13 |  |
| Hypertension [8] | 401, 402, 403, 404, 405 | I10, I11, I12, I13, I15 |  |
| (Other) Mental Illnesses | 291, 292, 295, 297, 298, 299, 301, 302, 303, 304, 305, 306, 307, 313, 314, 315, 319 | F04, F050, F058, F059, F060, F061, F062, F063, F064, F07, F08, F10, F11, F12, F13, F14, F15, F16, F17, F18, F19, F20,  F21, F22, F23, F24, F25, F26, F27, F28, F29, F340, F35, F36,  F37, F430, F439, F453, F454, F458, F46, F47, F49, F50, F51,  F52, F531, F538, F539, F54, F55, F56, F57, F58, F59, F60,  F61, F62, F63, F64, F65, F66, F67, F681, F688, F69, F70,  F71, F72, F73, F74, F75, F76, F77, F78, F79, F80, F81, F82,  F83, F84, F85, F86, F87, F88, F89, F90, F91, F92, F931,  F932, F933, F938, F939, F94, F95, F96, F97, F98 |  |
| Mood, anxiety, depression and other nonpsychotic disorders | 296, 300, 309, 311 | F30, F31, F32, F33, F34 (excl. F34.0), F38, F39, F40, F41,  F42, F43.1, F43.2, F43.8, F44, F45.0, F45.1, F45.2, F48,  F53.0, F68.0, F93.0, F99 |  |
| Osteoporosis | 733 | M81, M82 |  |
| Renal failure | 403, 404, 584, 585, 586, v451 | N17, N18, N19, T82.4, Z49.2, Z99.2 |  |
| Stroke (excluding transient ischemic attack) | 430, 431, 432, 434, 436 | I60I64 |  |

**Notes**: All available health administrative data (OHIP, DAD, ODB) prior to index is used to ascertain disease status, with the exception of AMI (1 year prior to index), Cancer (2 years), Mood Disorder (2 years) and Other Mental Illnesses (2 years) as these conditions are considered episodic. AMI, Asthma, COPD, CHF, Dementia, Diabetes, Hypertension, IBD, and Rheumatoid Arthritis are based on validated case algorithms/ ICES cohorts (see 1-9 below, respectively). All other conditions required at least one diagnosis code recorded in acute care (DAD) or two diagnosis codes recorded in physician billings (OHIP) within a two-year period. ODB prescription drug records are not available for the majority of persons under the age of 65. DAD=Discharge Abstract Database; ICD = International Classification of Disease; ODB = Ontario Drug Benefit Claims database; OHIP = Ontario Health Insurance Plan Claims Database.

References:

1. Austin PC, Daly PA, Tu JV. A multicenter study of the coding accuracy of hospital discharge administrative data for patients admitted to cardiac care units in Ontario. Am Heart J 2002;144:290–6.

2. Widdifield J, Bernatsky S, Paterson JM, Tu K, Ng R, Thorne JC, et al. Accuracy of Canadian health administrative databases in identifying patients with rheumatoid arthritis: a validation study using the medical records of rheumatologists. Arthritis Care Res 2013; 65(10): 1582-91.

3. Gershon AS, Wang C, Guan J, Vasilevska-Ristovska J, Cicutto L, To T. Identifying patients with physician-diagnosed asthma in health administrative databases. Can Respir J 2009;16:183–8.

4. Schultz SE, Rothwell DM, Chen Z, Tu K. Identifying cases of congestive heart failure from administrative data: a validation study using primary care patient records. Chronic Dis Inj Canada 2013;33:160–6.

5. Gershon AS, Wang C, Guan J, Vasilevska-Ristovska J, Cicutto L, To T. Identifying Individuals with Physician Diagnosed COPD in Health Administrative Databases. COPD 2009;6:388–94.

6. Jaakkimainen RL, Bronskill SE, Tierney MC, Herrmann N, Green D, Young J, et al. Identification of Physician-Diagnosed Alzheimer’s Disease and Related Dementias in Population-Based Administrative Data: A Validation Study Using Family Physicians’ Electronic Medical Records. J Alzheimers Dis 2016;54(1):337–49.

7. Hux JE, Ivis F, Flintoft V, Bica A. Diabetes in Ontario: Determination of prevalence and incidence using a validated administrative data algorithm. Diabetes Care 2002;25:512–6.

8. Tu K, Campbell NR, Chen ZL, Cauch-Dudek KJ, McAlister FA. Accuracy of administrative databases in identifying patients with hypertension. Open Med 2007;1:e18–26.

9. Benchimol EI, Guttmann A, Mack DR, Nguyen GC, Marshall JK, Gregor JC, et al. Validation of international algorithms to identify adults with inflammatory bowel disease in health administrative data from Ontario, Canada, J Clin Epidemiol 2014;67(8):887-96.

**Additional File Table 3**: Procedure/Intervention Codes.

| **Procedure/Intervention** | **Associated Database and Codes** |
| --- | --- |
| Dialysis | Discharge database: DAD  Code: 1.PZ.21- |
| Invasive Mechanical Ventilation | Discharge database: DAD  Code: 1.GZ.31.CRND, 1.GZ.31.GPND, 1.GZ.31.CA- |
| Noninvasive Ventilation | Discharge database: DAD  Code: 1.GZ.31.CB-, 1.GZ.31.JA- |
| Bronchoscopy | Discharge database: DAD  Code: 2.GM.70- |
| Percutaneous Coronary Intervention (PCI) | Discharge database: DAD  Codes: 1IJ50-, 1IJ54-, 1IJ57GQ- |
| Feeding tube | Discharge database: DAD  Code: 1.NF.53- |
| Blood transfusion | Discharge database: DAD  Code: 1.LZ.19- |
| Cardiopulmonary Resuscitation (CPR) | Discharge database: DAD  Code: 1.HZ.30- |
| Defibrillation | Discharge database: DAD  Code: 1.HZ.09- |

**Additional File Table 4**: Palliative Care Codes.

| Palliative Care | Discharge database: DAD  Code: Z515, patserv = ‘58’ |
| --- | --- |

**Additional File Table 5**: Severity of cognitive and functional impairment in patients with dementia.

| **Variable** | **Definition** |
| --- | --- |
| Severity of cognitive impairment  Minimal  Moderate  Severe  Missing | Based on Cognitive Performance Scale [CPS] score  Intact or borderline intact  Mild impairment or moderate impairment or moderate/severe impairment  Severe impairment or very severe impairment  Missing data |
| ADL scale  Independent  Minimal assistance  Extensive assistance  Dependent  Missing | Independent  Supervision or limited impairment  Extensive assistance required (I+II)  Dependent or total dependence  Missing Data |
| IADL scale  No difficulty  Minimal difficulty Significant difficulty  Missing | No difficulty in any of three IADLs  Some difficulty in one, two, or all three IADLs  Great difficulty in one, two, or all three IADLs  Missing Data |
| CHESS scale  No health instability  Mild-moderate instability  Severe health instability  Missing | Score 0/5  Score 1-3/5  Score 4-5/5  Missing Data |

Notes: ADL=activities of daily living; IADL=instrumental activities of daily living; CHESS=changes in health, end-stage disease, and signs and symptoms.

**Additional File Table 6**: Hierarchy approach for discharge disposition of those who survived to discharge.

1. **Discharged to home with homecare** (defined as: at least one homecare service within 21 days of discharge, as identified via the Home Care Database)

2. **Discharged to rehabilitation or complex continuing care** (defined as: at least one admission to complex continuing care or a rehabilitation bed, as identified via the Continuing Care Reporting System or the National Rehabilitation Reporting System, respectively)

3. **Discharged to a long-term care facility** (defined as: at least one admission to long-term care within 2 days of discharge, as identified via the Continuing Care Reporting System – Long-term Care)

4. **Died in hospital** (defined as: death date occurring on or prior to discharge date, as identified via the Discharge Abstract Database)

5. **Discharged to home without homecare** (if patient did not meet other four criteria above, then included in this group)

*In cases where a patient is eligible for more than one disposition category, the institution with the first date is selected as priority. In cases where the dates overlap, the following hierarchy is used: death > complex continuing care/rehabilitation > long-term care facility > homecare

**Additional File Table 7:** Multivariate logistic regression analysis for survival in patients with dementia admitted to ICU and non-ICU hospital settings.

| **Variables** | **OR** | **Confidence Interval** | **P value** |
| --- | --- | --- | --- |
| Index admission  Non-ICU hospital settings  ICU | Reference  0.33 | Reference  0.31 – 0.34 | <.0001 |
| Age  65-75  76-85  86-95  95+ | Reference  0.69  0.47  0.31 | Reference  0.65 – 0.74  0.44 – 0.50  0.28 – 0.34 | <.0001  <.0001  <.0001 |
| Sex  Male  Female | Reference  1.34 | Reference  1.29 – 1.39 | <.0001 |
| Income  Lowest  Low  Middle  High  Highest | Reference  0.97  0.98  1.00  1.02 | Reference  0.91 – 1.02  0.92 – 1.04  0.94 – 1.07  0.95 – 1.08 | 0.23  0.50  0.97  0.65 |
| Top 5 comorbidities  Hypertension  Diabetes  Cancer  CHF  CAD | 1.27  1.26  1.12  0.85  1.29 | 1.22 – 1.33  1.21 – 1.32  1.07 – 1.17  0.81 – 0.90  1.21 – 1.38 | <.0001  <.0001  <.0001  <.0001  <.0001 |
| Charlson comorbidity score  0-2  3+ | Reference  0.39 | Reference  0.37 – 0.41 | <.0001 |
| Number of hospital admissions pre-index admission  0  1+ | Reference  0.93 | Reference  0.90 – 0.96 | <.0001 |
| Number of ED visits pre-index admission  0  1+ | Reference  1.04 | Reference  1.02 – 1.05 | <.0001 |

**Notes**: Odds Ratios (OR) were calculated for each variable. Index hospital admission, age 65-75, male sex, lowest income quintile, and Charlson comorbidity score of 0-2 were used as the reference comparison group. For the top 5 medical comorbidities, the reference for each comorbidity was not having that specific comorbidity. Top 5 medical comorbidities were determined by using highest prevalence of medical conditions (excluding mood and mental health) from patient demographics (Table 1 in main manuscript). 95% confidence intervals are displayed. ED=emergency department.

**Additional File Table 8:** Multivariate logistic regression analysis for ICU admission in patients with dementia.

| **Variables** | **OR** | **Confidence Interval** | **P value** |
| --- | --- | --- | --- |
| Severity of cognitive impairment  Minimal  Moderate-severe | Reference  0.78 | Reference  0.73 – 0.82 | <.0001 |
| Age  65-75  76-85  86-95  95+ | Reference  0.70  0.49  0.32 | Reference  0.64 – 0.75  0.46 – 0.54  0.27 – 0.38 | <.0001  <.0001  <.0001 |
| Sex  Male  Female | Reference  0.89 | Reference  0.83 – 0.94 | <.0001 |
| Income  Lowest  Low  Middle  High  Highest | Reference  0.91  1.01  0.91  0.89 | Reference  0.84 – 0.99  0.93 – 1.10  0.84 – 1.00  0.81 – 0.97 | 0.02  0.76  0.04  0.009 |
| Top 5 comorbidities  Hypertension  Diabetes  Cancer  CHF  CAD | 1.02  1.07  0.90  1.28  1.13 | 0.96 – 1.08  1.01 – 1.14  0.84 – 0.96  1.18 – 1.38  1.03 – 1.23 | 0.55  0.03  0.003  <.0001  0.008 |
| Charlson comorbidity score  0-2  3+ | Reference  1.67 | Reference  1.56 – 1.79 | <.0001 |
| Number of hospital admissions pre-index admission  0  1+ | Reference  1.09 | Reference  1.05 – 1.13 | <.0001 |
| Number of ED visits pre-index admission  0  1+ | Reference  0.93 | Reference  0.91 – 0.94 | <.0001 |

**Notes**: This subset is for patients with dementia admitted to the ICU who had an RAI assessment completed. Odds Ratios (OR) were calculated for each variable. Minimal dementia severity, age 65-75, male sex, lowest income quintile, and Charlson comorbidity score of 0-2 were used as the reference comparison group. For the top 5 medical comorbidities, the reference for each comorbidity was not having that specific comorbidity. Top 5 medical comorbidities were determined by using highest prevalence of medical conditions (excluding mood and mental health) from patient demographics (Table 1 in main manuscript). 95% confidence intervals are displayed. ED=emergency department.

**Additional File Table 9:** Multivariate logistic regression analysis age- and sex-stratified for mortality in patients with dementia.

|  | **1-year Mortality** | |
| --- | --- | --- |
| **Age Category** | **Male** | **Female** |
| 65-75 | 33.8% | 32.1% |
| 76-85 | 42.6% | 39.3% |
| 86-95 | 55.8% | 49.4% |
| 95+ | 78.3% | 65.6% |

Notes: Mortality at one-year post admission is described as a proportion of all patients with dementia in the cohort, stratified by age and sex.

**Additional File Table 10:** Multivariate logistic regression analysis for mortality in patients with dementia admitted to ICU only.

| **Variables** | **OR** | **Confidence Interval** | **P value** |
| --- | --- | --- | --- |
| Age  65-75  76-85  86-95  95+ | Reference  1.34  1.87  2.96 | Reference  1.23 – 1.45  1.72 – 2.03  2.50 – 3.49 | <0.0001  <0.0001  <0.0001 |
| Sex  Male  Female | Reference  0.88 | Reference  0.83 – 0.94 | <0.0001 |
| Income  Lowest  Low  Middle  High  Highest | Reference  1.01  0.98  0.92  0.97 | Reference  0.94 – 1.10  0.90 – 1.06  0.84 – 1.00  0.88 – 1.06 | 0.74  0.62  0.06  0.46 |
| Top 5 comorbidities  Hypertension  Diabetes  Cancer  CHF  CAD | 0.80  0.95  0.88  1.20  0.75 | 0.75 – 0.85  0.89 – 1.01  0.82 – 0.94  1.12 – 1.30  0.69 – 0.82 | <0.0001  0.12  0.0002  <0.0001  <0.0001 |
| Charlson comorbidity score  0-2  3+ | Reference  1.66 | Reference  1.56 – 1.78 | <0.0001 |
| Number of hospital admissions pre-index admission  0  1+ | Reference  1.09 | Reference  1.04 – 1.13 | <0.0001 |
| Number of ED visits pre-index admission  0  1+ | Reference  1.00 | Reference  0.99 – 1.02 | 0.62 |

**Notes**: Odds Ratios (OR) were calculated for each variable. Index hospital admission, age 65-75, male sex, lowest income quintile, and Charlson comorbidity score of 0-2 were used as the reference comparison group. For the top 5 medical comorbidities, the reference for each comorbidity was not having that specific comorbidity. Top 5 medical comorbidities were determined by using highest prevalence of medical conditions (excluding mood and mental health) from patient demographics (Table 1 in main manuscript). 95% confidence intervals are displayed. ED=emergency department.

**Additional File Table 11:** Multivariate logistic regression analysis for mortality in patients with dementia admitted to ICU only with RAI assessment available.

| **Variables** | **OR** | **Confidence Interval** | **P value** |
| --- | --- | --- | --- |
| Severity of cognitive impairment  Minimal  Moderate-severe | Reference  1.35 | Reference  1.25 – 1.45 | <0.0001 |
| Age  65-75  76-85  86-95  95+ | Reference  1.18  1.47  2.35 | Reference  1.06 – 1.31  1.32 – 1.64  1.91 – 2.89 | 0.003  <0.0001  <0.0001 |
| Sex  Male  Female | Reference  0.85 | Reference  0.79 – 0.92 | <0.0001 |
| Income  Lowest  Low  Middle  High  Highest | Reference  1.05  0.98  0.93  0.99 | Reference  0.95 – 1.16  0.88 – 1.09  0.83 – 1.04  0.87 – 1.11 | 0.35  0.66  0.21  0.81 |
| Top 5 comorbidities  Hypertension  Diabetes  Cancer  CHF  CAD | 0.86  0.93  0.96  1.17  0.85 | 0.79 – 0.93  0.86 – 1.01  0.87 – 1.05  1.06 – 1.29  0.76 – 0.96 | 0.0001  0.08  0.33  0.008  0.002 |
| Charlson comorbidity score  0-2  3+ | Reference  1.50 | Reference  1.37 – 1.63 | <0.0001 |
| Number of hospital admissions pre-index admission  0  1+ | Reference  1.03 | Reference  0.98 – 1.08 | 0.23 |
| Number of ED visits pre-index admission  0  1+ | Reference  0.99 | Reference  0.97 – 1.02 | 0.56 |

**Notes**: This subset is for patients with dementia admitted to the ICU who had an RAI assessment completed. Odds Ratios (OR) were calculated for each variable. Minimal dementia severity, age 65-75, male sex, lowest income quintile, and Charlson comorbidity score of 0-2 were used as the reference comparison group. For the top 5 medical comorbidities, the reference for each comorbidity was not having that specific comorbidity. Top 5 medical comorbidities were determined by using highest prevalence of medical conditions (excluding mood and mental health) from patient demographics (Table 1 in main manuscript). 95% confidence intervals are displayed. ED=emergency department.

**Additional File Table 12:** Multivariate logistic regression analysis for mortality in patients with dementia admitted to non-ICU hospital settings only.

| **Variables** | **OR** | **Confidence Interval** | **P value** |
| --- | --- | --- | --- |
| Age  65-75  76-85  86-95  95+ | Reference  1.44  2.21  3.51 | Reference  1.39 – 1.50  2.13 – 2.29  3.34 – 3.70 | <0.0001  <0.0001  <0.0001 |
| Sex  Male  Female | Reference  0.79 | Reference   - 1. – 0.81 | <0.0001 |
| Income  Lowest  Low  Middle  High  Highest | Reference  1.01  0.99  1.02  0.99 | Reference  0.99 – 1.04  0.96 – 1.02  0.99 – 1.05  0.95 – 1.02 | 0.35  0.35  0.30  0.38 |
| Top 5 comorbidities  Hypertension  Diabetes  Cancer  CHF  CAD | 0.76  0.88  0.98  1.28  0.85 | 0.74 – 0.77  0.86 – 0.90  0.96 – 1.01  1.25 – 1.32  0.82 – 0.88 | <0.0001  <0.0001  0.18  <0.0001  <0.0001 |
| Charlson comorbidity score  0-2  3+ | Reference  1.97 | Reference  1.92 – 2.02 | <0.0001 |
| Number of hospital admissions pre-index admission  0  1+ | Reference  1.09 | Reference  1.08 – 1.11 | <0.0001 |
| Number of ED visits pre-index admission  0  1+ | Reference  0.98 | Reference  0.98 – 0.99 | <0.0001 |

**Notes**: Odds Ratios (OR) were calculated for each variable. Index hospital admission, age 65-75, male sex, lowest income quintile, and Charlson comorbidity score of 0-2 were used as the reference comparison group. For the top 5 medical comorbidities, the reference for each comorbidity was not having that specific comorbidity. Top 5 medical comorbidities were determined by using highest prevalence of medical conditions (excluding mood and mental health) from patient demographics (Table 1 in main manuscript). 95% confidence intervals are displayed. ED=emergency department.

**Additional File Table 13:** Multivariate logistic regression analysis for mortality in patients with dementia admitted to non-ICU hospital settings only with RAI assessment available.

| **Variables** | **OR** | **Confidence Interval** | **P value** |
| --- | --- | --- | --- |
| Severity of cognitive impairment  Minimal  Moderate-severe | Reference  1.66 | Reference  1.62 – 1.71 | <0.0001 |
| Age  65-75  76-85  86-95  95+ | Reference  1.35  1.82  2.61 | Reference  1.28 – 1.41  1.74 – 1.91  2.46 – 2.77 | <0.0001  <0.0001  <0.0001 |
| Sex  Male  Female | Reference  0.77 | Reference  0.75 – 0.79 | <0.0001 |
| Income  Lowest  Low  Middle  High  Highest | Reference  1.02  1.02  1.05  1.04 | Reference  0.99 – 1.06  0.98 – 1.06  1.01 – 1.09  1.00 – 1.08 | 0.19  0.34  0.02  0.07 |
| Top 5 comorbidities  Hypertension  Diabetes  Cancer  CHF  CAD | 0.83  0.90  1.06  1.28  0.93 | 0.81 – 0.85  0.86 – 0.92  1.03 – 1.09  1.24 – 1.33  0.89 – 0.97 | <0.0001  <0.0001  0.0002  <0.0001  0.0003 |
| Charlson comorbidity score  0-2  3+ | Reference  1.66 | Reference  1.60 – 1.71 | <0.0001 |
| Number of hospital admissions pre-index admission  0  1+ | Reference  1.05 | Reference  1.03 – 1.07 | <0.0001 |
| Number of ED visits pre-index admission  0  1+ | Reference  0.98 | Reference  0.97 – 0.99 | <0.0001 |

**Notes**: This subset is for patients with dementia admitted to non-ICU settings who had an RAI assessment completed. Odds Ratios (OR) were calculated for each variable. Minimal dementia severity, age 65-75, male sex, lowest income quintile, and Charlson comorbidity score of 0-2 were used as the reference comparison group. For the top 5 medical comorbidities, the reference for each comorbidity was not having that specific comorbidity. Top 5 medical comorbidities were determined by using highest prevalence of medical conditions (excluding mood and mental health) from patient demographics (Table 1 in main manuscript). 95% confidence intervals are displayed. ED=emergency department.
